# Prediagnostic Intervals in Retinoblastoma: Experience at an Oncology Center in Brazil

**DOI:** 10.1200/JGO.2016.005595

**Published:** 2016-10-12

**Authors:** Clarissa Campolina de Sá Mattosinho, Nathalia Grigorovski, Evandro Lucena, Sima Ferman, Anna Tereza Miranda Soares de Moura, Arlindo Freire Portes

**Affiliations:** **All authors:** Instituto Nacional de Câncer; Universidade Estácio de Sá; and Universidade Estadual do Rio de Janeiro, Rio de Janeiro, Brazil.

## Abstract

**Purpose:**

Retinoblastoma is the most common intraocular malignancy of childhood. In most cases, parents are the first to notice leukocoria and other symptoms before undergoing a prolonged period of stress before diagnosis. The purpose of this study was to determine prediagnostic intervals of patients with retinoblastoma at an oncology tertiary center (Instituto Nacional de Cancer) in Rio de Janeiro, Brazil, and relate them to stage at diagnosis, eye salvage, and survival.

**Methods:**

Parents or caregivers of children with retinoblastoma registered between January 2006 and September 2013 were interviewed using a semistructured individually applied questionnaire, concerning their trajectory before registration.

**Results:**

Out of 76 patients, 39 (51%) were girls, 52 (68%) had unilateral retinoblastoma, and 24 (32%) had bilateral retinoblastoma, totaling 100 affected eyes. The most common stage of diagnosis was the intraocular group, with 63 (83%) patients; nine (12%) were extraocular, and four (5%) had metastatic disease. During the follow-up time of 37 ± 24.5 months, 10 (13%) patients died and 70 (70%) eyes were enucleated. Mean family interval was 1.6 ± 2.6 months, mean medical interval was 5.0 ± 6.2 months, mean referral interval was 0.2 ± 1.4 months, and mean overall interval was 7.1 ± 6.9 months. In univariate analysis, age at diagnosis, maternal education, medical interval, and overall interval were significantly related to advanced stage at diagnosis and survival. In multivariate analysis, maternal education and medical interval were significantly related to advanced stage at diagnosis and survival. No variables affected eye salvage.

**Conclusion:**

Medical interval was responsible for 70% of the overall interval; therefore, programs or campaigns targeting retinoblastoma early diagnosis should focus emphasize in medical awareness.

## INTRODUCTION

Retinoblastoma is the most common intraocular malignancy of childhood, usually first detected by parents.^1^ Population-based studies show a higher incidence of retinoblastoma in some countries, such as Brazil,^2^ where approximately 400 children are diagnosed each year.^3^

In developed countries, retinoblastoma is usually diagnosed in early intraocular stages, and the survival rate is > 90%.^4,5^ However, in developing countries, approximately 40% of the children affected still die at young age of metastatic disease.^6,7^

In developing countries, the diagnosis of retinoblastoma is usually made at advanced stages, lowering ocular and patient survival.^8^ The survival of patients with retinoblastoma in these countries is influenced by socioeconomic and cultural factors, resulting in late diagnosis. There is also a shortage of modern treatments in these countries.^9,10^

Early detection permits the use of treatments that preserve the eyes, minimizing morbidity.^8,11^ When there is lack of knowledge about the disease, the time to make the diagnosis is longer, delaying referral to a specialized treatment center. This allows the disease to become more advanced and reduces the chances for healing.^12^

Prognosis of most pediatric tumors is related to tumor biology^13^; in retinoblastoma, late diagnosis is a major determinant for disease dissemination.^9,14^ At least 50% of the 5,000 to 8,000 new cases worldwide will present symptoms of extraocular disease.^7^

The outcome of patients with retinoblastoma in developing countries is highly influenced by the lack of early diagnosis.^15^ Therefore, understanding the particularities of prediagnostic intervals is crucial to launch campaigns focused on the real sources of delays. The purpose of this study was to determine the duration of segments composing prediagnostic intervals of patients with retinoblastoma and relate those intervals with advanced stage at diagnosis, eye salvage, and patient survival.

## METHODS

Parents or caregivers of children with retinoblastoma treated at an oncology tertiary center (Instituto Nacional de Câncer) in Rio de Janeiro, Brazil, between January 2006 and September 2013 were interviewed about their trajectory before registration. Most interviews were conducted taking advantage of previously scheduled consultations in the ocular oncology outpatient unit. Patients who did not have booked consultations throughout the data collection period were invited to participate by telephone. Three attempts at contact were made at different times and dates using all phone numbers available from the patient chart. Although some invitations were conducted by telephone, all interviews were performed face to face. Respondents were always parents or the child’s legal caregiver who previously signed the consent form. There was no difference in procedure according to the parent’s educational level. The interview did not require reading or writing abilities, because all questions were orally spoken and answers were transcribed by the investigator himself. In cases of deceased patients, all interviews were conducted after the child’s death. Those parents were also invited to participate in the study by telephone or during scheduled appointments for siblings who were still being screened for retinoblastoma.

The report of prediagnostic history was recorded in a uniform format using a semistructured questionnaire. The only open question was relative to first perceived sign, the answer to which was literally copied and then categorized according to the most frequent signs cited in the literature on this subject as leukocoria, strabismus, red eye, low vision, and others.^16^

Respondents were asked about the sequence of events from the initial moment when someone noticed something different in the child’s eyes until registration. Socioeconomic data were obtained from all patients during the interview, assessing place of residency, maternal education (years spent in school), number of people per household, and private health insurance.

According to the Aarhus statement regarding prediagnostic patient pathways, emphasis was given to the initial ocular symptoms noted and prediagnostic intervals. Diagnosis date was defined as the registration date, using criteria suggested by the European Network of Cancer Registries.^17^ Prediagnostic intervals were defined by such decisive dates as the moment of perception of first symptom, first consultation after perception, and date of referral to the oncology care institution.

Prediagnostic intervals definitions used in this study are as follows: Family interval is the time between first symptom noticed and first consultation with a doctor. Medical interval is the time between the first consultation and referral to the oncology tertiary center. Referral interval is the time between referral and registration in the oncology tertiary center. Overall interval is the time between perception of the first symptoms and date of registration (family plus medical plus referral). The following clinical parameters were taken from patient charts: laterality, date of birth, date of registration, stage at registration, treatment information, and eye and patient survival.

The International Retinoblastoma Staging System^18^ (0, I, II, III, IV) was used and categorized for statistical analyses as follows: intraocular disease (stage 0, I, II), extraocular disease (stage III), metastatic disease (stage IV). For some comparisons we used the term advanced stage at diagnosis (stage III plus stage IV). For each affected eye we used the International Classification of Retinoblastoma grouping system (A, B, C, D, E).

Statistical analysis was performed using Epi Info 7. Bivariate and multivariate analysis consisted of logistic regression, using a 5% significance level and 95% CIs. The informed consent was provided to parents or caregivers of the children surveyed. The study was accepted by the local ethics committee.

## RESULTS

Out of 94 registered patients with retinoblastoma during the study period, eight patients received initial treatment outside of our institution and were excluded, one parent refused to participate, and nine parents could not be reached through telephone numbers provided in patient charts. Parents or caregivers of 76 patients were available and accepted the invitation to the interview.

Table 1 shows descriptive statistics of all 76 patients, and Table 2 shows the extent of prediagnostic intervals. Leukocoria was cited in association with other signs in six cases, and strabismus was associated with red eye in one case.

**Table 1:**
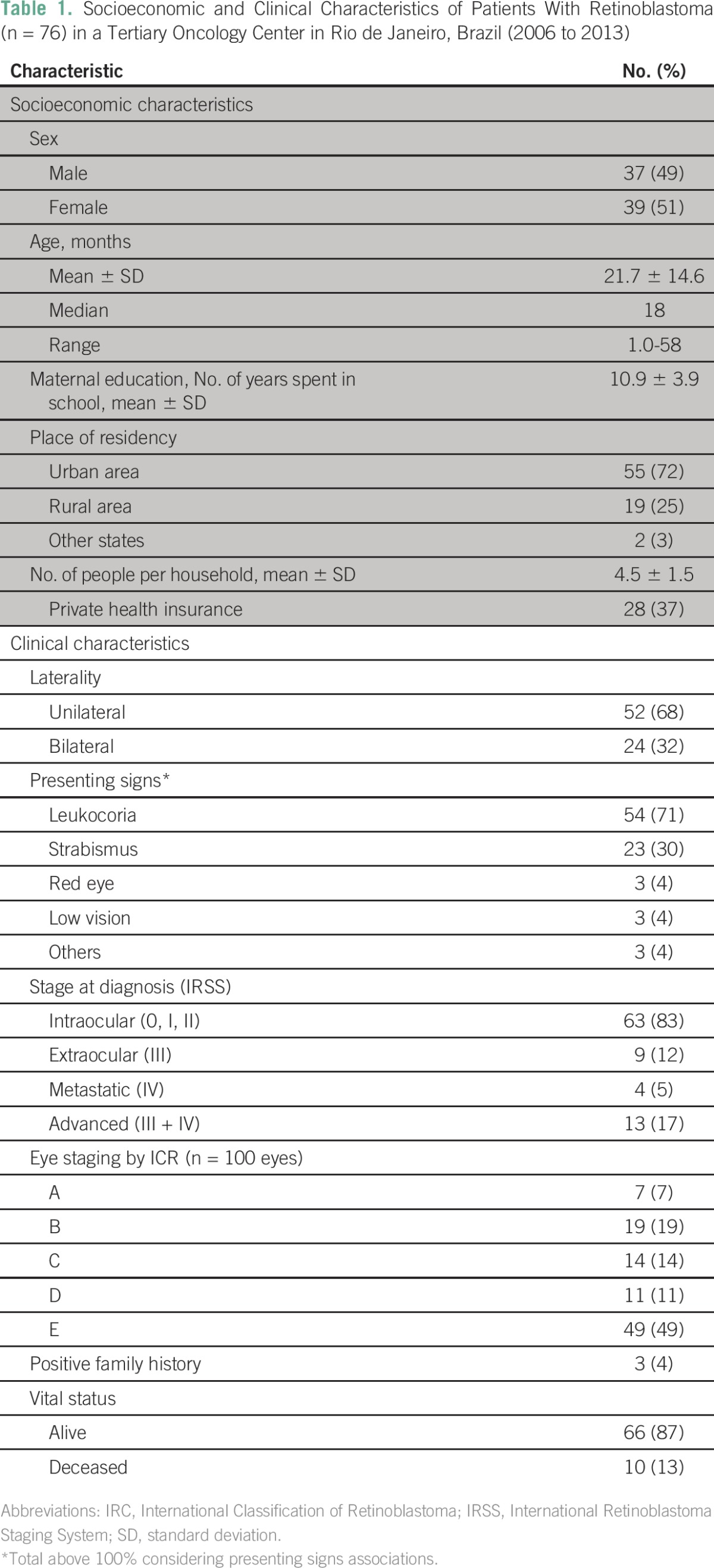
– Socioeconomic and Clinical Characteristics of Patients With Retinoblastoma (n = 76) in a Tertiary Oncology Center in Rio de Janeiro, Brazil (2006 to 2013)

**Table 2:**
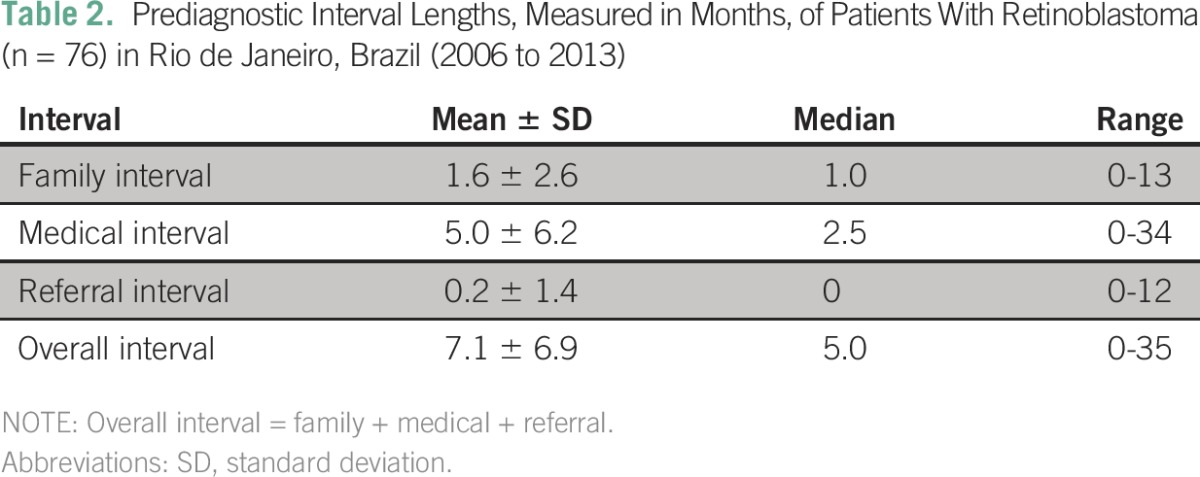
– Prediagnostic Interval Lengths, Measured in Months, of Patients With Retinoblastoma (n = 76) in Rio de Janeiro, Brazil (2006 to 2013)

In bivariate analysis (Table 3), mean maternal education was significantly related to advanced stage at diagnosis (*P* = .016) and survival (*P* = .003). Other than maternal education, none of the socioeconomic characteristics (people per household, place of residency, coverage by private health insurance) carried a significant difference with outcomes (advanced stage at diagnosis, enucleation, and survival). Medical interval, overall interval, and age at diagnosis were significantly related to advanced stage at diagnosis and survival. Enucleation was not significantly affected by any of those variables.

**Table 3:**
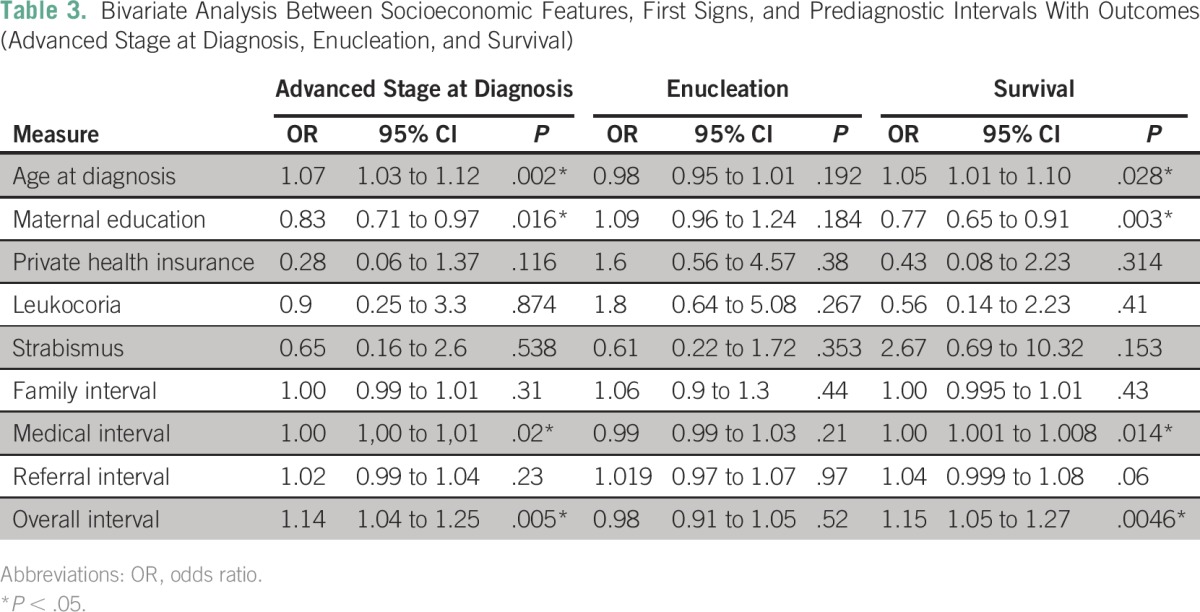
– Bivariate Analysis Between Socioeconomic Features, First Signs, and Prediagnostic Intervals With Outcomes (Advanced Stage at Diagnosis, Enucleation, and Survival)

Multivariate analysis is shown in Table 4 by logistic regression. Maternal education was selected by virtue of significant association in bivariate analysis. All prediagnostic intervals were also included, because they were part of the study purpose. Age at diagnosis and overall interval were excluded from this analysis because those variables were highly correlated with other interval variables (family, medical, and referral intervals), and using them all together could produce collinearity, with a consequent loss of statistical significance. Medical interval and maternal education were significantly related to advanced stage at diagnosis and survival.

**Table 4:**
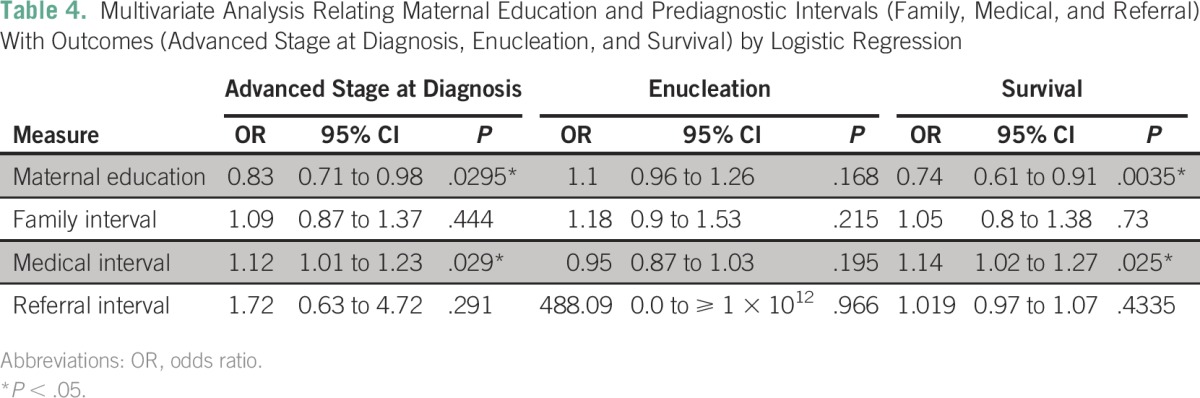
– Multivariate Analysis Relating Maternal Education and Prediagnostic Intervals (Family, Medical, and Referral) With Outcomes (Advanced Stage at Diagnosis, Enucleation, and Survival) by Logistic Regression

Out of 100 affected eyes, 70% were enucleated. Among bilateral cases (n = 24), three patients had bilateral enucleations, four patients had both eyes preserved, and 17 patients had one eye enucleated. Among unilateral cases (n = 52), 47 patients were enucleated and only five patients had the eye preserved. All 10 deaths (13%) were caused by disease progression or relapses; there was no toxic death. Mean interval from diagnosis to interview was 3.2 ± 2.2 years (range, 0 to 7 years; median, 3 years). The mean follow-up time was 37 ± 24.5 months.

## DISCUSSION

Prediagnostic intervals of pediatric cancers have been regularly reported for more than 50 years; they are a source of sorrow for physicians and parents,^14^ differing widely between tumor types. Determinants of prediagnostic intervals, such as parental attention, health care structure, and physician awareness, are difficult to assess.^14^

A systematic review of 98 papers about prediagnostic intervals in pediatric malignancies pointed out the often false general belief that a long delay before diagnosis always leads to worse prognosis.^14^ Brasme et al^14^ established that delayed diagnosis was associated with poor outcome for retinoblastoma and possibly for leukemia, nephroblastoma, and rhabdomyosarcoma. Besides those, pediatric cancer prediagnosis interval lengths depend more on the tumor’s biology than on parental or medical factors. Median overall interval revealed a wide variation among tumor types, from approximately 3 weeks in leukemia and neuroblastoma to approximately 5 to 6 weeks in lymphoma and rhabdomyosarcoma, and the median overall interval was 13 weeks for soft-tissue sarcomas.^14^

Even though recognizing that long diagnostic delay is not necessarily associated with poor outcomes, it might be a relief for family and doctors.^14^ Delayed diagnosis is a source of regret for physicians and parents and a prominent cause of malpractice lawsuits.^14^ Therefore, the authors suggested suitable ways to achieve early diagnosis, including taking into account parent complaints, never ignoring persisting symptoms, and thoroughly investigating signs or symptoms that are not comprehended.^14^

The term delay in diagnosis is commonly used to describe an undefined period between symptom onset and definitive diagnosis. This term has negative connotations, implies criticism, and is often used to assign blame.^19,20^ Authors frequently used an arbitrary time point defined by their observations to qualify the moment when a delay becomes unacceptable or excessive.^19^ Using more neutral terms, such as prediagnostic interval, is now advised.^20^ This period is typically subdivided into two components, the first one between onset of symptoms and first consultation and the second one between first consultation and diagnosis. This study also divided the second period into two parts: before and after referral to the oncology tertiary center.

Our study relied on parent recall of events that took place many months or even years before, possibly introducing a memory bias. However, most studies about prediagnostic intervals are based on chart information that may have missing information. Because parents were all personally interviewed, and events related to childhood cancer often cause deep family scars, the results may be more accurate than those extracted essentially from chart information. The systematic review previously mentioned about delayed diagnosis of pediatric cancer published in 2012^14^ found only two studies that included semistructured interviews with parents whose children had a diagnosis of cancer.^21,22^ In both studies, time to diagnosis was important for parents, independently of time between diagnosis and the interview. Parental experiences during this period may affect their reaction to diagnosis, treatment, and trust in the health care system.^14^

Although Brazil is a country with large continental distances and enormous geographical barriers, most patients (72.3%) were living within the metropolitan area of the city of Rio de Janeiro, the second most populous city in Brazil, with approximately 6.3 million inhabitants. Even those living in rural areas were not extremely isolated. The State of Rio de Janeiro is the fourth smallest state (in area) in Brazil, with 43,000 km^2^, and has good road infrastructure without any major geographic barriers.

Maternal education was associated with advanced stage at diagnosis and survival, corroborating previous studies establishing the association between maternal education and pediatric cancer prognosis.^23^ Other studies have shown a parental education association with late consultation in retinoblastoma.^10^ A recent systematic review of the survival of retinoblastoma in less-developed countries showed a relationship between socioeconomic indicators and infant health outcome.^8^

The potential association between health insurance and cancer diagnosis and outcomes has lately been the theme of increasing interest. Although this study did not find correlation on this topic, a recent paper suggested a higher rate of more advanced disease associated with nonprivate health insurance in patients with retinoblastoma.^24^

The median family interval was 4 weeks. A previous study held in a high-income country described a median family interval of 2.5 weeks.^25^ It is certain that health systems from middle-income countries such as Brazil differ from high-income health systems and likely contribute to disparities in family interval. Nevertheless, the results were corroborated by DerKinderen et al,^26^ who described a similar family interval. It is noteworthy that family interval did not significantly alter prognosis.

The Brazilian study of Erwenne and Franco^9^ in 1989 was a pioneer in demonstrating a direct correlation between delay in diagnosis and extraocular disease and poor prognosis in retinoblastoma. Another Brazilian study, by Antoneli et al,^12^ found, for the periods of 1986 to 1990 and 1991 to 1995, a mean overall interval of 7.5 months and 5.3 months, respectively. However, the results for overall interval remain compatible with those found in the 1980s, which indicates the challenges of increasing the awareness of a rare disease in a vast and highly populated country like Brazil. A national campaign for the early diagnosis of retinoblastoma began in September 2002, offering educational material for the population, primary care staff, and ophthalmologists. A nationwide toll-free number was also available for information, and a video was broadcast on public television channels.^27^

The mean overall interval was 7 months and showed a statistically significant relation with advanced stage at diagnosis (*P* < .005) and survival (*P* < .0046). The median overall interval (5 months) was more than twice as long as that described by Goddard et al^25^ and Wallach et al.^28^ This drastic disparity in the overall interval between developing and developed countries might be one of the reasons for worse outcomes in poorer countries.

Medical interval was accountable for the longest part of the overall interval, with a 5-month average. It is alarming to acknowledge that medical interval is responsible for almost total extension (77%) of overall interval. Other studies showed that medical interval accounted for 23% of overall interval,^14^ and medical delays ≥ 1 week led to significantly higher rates of death and blindness.^26^ Physicians have a tendency to trivialize some symptoms, especially when the disease is easily forgotten because of its rarity.^29^ It seems that there is difficulty in interpreting symptoms correctly.^26^ In the multivariate analysis, medical interval was the only interval related significantly to prognosis (advanced stage at diagnosis and survival). According to DerKinderen et al,^26^ parents usually notice the tumor in its earlier stages when it has not yet reached the critical size, but a physician’s delay may allow tumor growth beyond critical size, leading to blindness and death.

The study revealed that early diagnosis ensures a more favorable initial staging. The main evidence is that medical interval must be the focus to reduce overall interval. Programs targeting mainly parent education about retinoblastoma may not be successful in all settings, because families did not take long to notice the first signs and seek medical help. Physicians were responsible for the longer part of prediagnostic intervals. Apparently, there is a lack of knowledge and awareness about retinoblastoma among first-contact physicians in the Brazilian health system, especially in Rio de Janeiro. This problem was also described in a Mexican study by Leal-Leal et al^30^ showing that medical students, just weeks from starting a primary care program, were not able to make a timely retinoblastoma diagnosis.^30^

No association was found between prediagnostic intervals and globe salvage. This is consistent with other studies, such as those of Abramson et al^1^ and Butros et al,^31^ who did not related prediagnostic intervals with ocular morbidity in retinoblastoma. Recently, new therapeutic modalities such as intra-arterial chemotherapy are being implemented, enhancing globe salvage rates.

Even though early diagnosis in retinoblastoma depends also on socioeconomic factors intrinsic to each country, actions to promote early diagnosis and referral may have impact especially in low-income countries.^6^ The partnership, known as twinning, between institutions from developed countries providing support to build and train local teams, associated with monetary funding, is essential to achieve better outcomes in less-developed countries.^32^ Twinning experiences have proven successful worldwide, with several examples of improvement in outcomes with cancer worldwide.^32-35^ Nevertheless, the partnership between high-income countries and upper-middle–income countries, such as Brazil, has more limited information, and even less in the retinoblastoma field.^36^ A successful collaboration program was created in 1995 between a tertiary care, public pediatric hospital in Argentina (upper-middle–income country) and a major specialized program in a world leading referral center for retinoblastoma in the United States.^36^ This partnership showed impressive results in Argentina and also in other countries, because it became a training center for Latin America specialists, with partial support from the Fund for Ophthalmic Knowledge.^36^ This partnership allowed the implementation of intra-arterial chemotherapy in our center, considerably improving the eye salvage rate. If programs like this, between Argentina and the United States, were implemented in Brazil, other aspects of retinoblastoma care, such as diagnostic process and medical awareness, would undoubtedly be improved.

In conclusion, even though the study was based on a limited number of patients from a single institution, medical education about retinoblastoma seems to be an important key to facilitating the recognition of first symptoms and shortening the overall interval. It is important to note that medical interval is highly influenced by health system organization and structure. The results should not be seen as a report to cast blame but as a guide to implement campaigns and resources in medical education concerning retinoblastoma.
